# Duodenal Pseudomelanosis: A Literature Review

**DOI:** 10.3390/diagnostics11111974

**Published:** 2021-10-24

**Authors:** Gianluca Lopez, Marianna D’Ercole, Stefano Ferrero, Giorgio Alberto Croci

**Affiliations:** 1 Pathology Unit, Fondazione IRCCS Ca’ Granda Ospedale Maggiore Policlinico, 20122 Milan, Italy; stefano.ferrero@unimi.it (S.F.); giorgio.croci@unimi.it (G.A.C.); 2 School of Pathology, University of Milan, 20122 Milan, Italy; marianna.dercole@unimi.it; 3 Department of Biomedical, Surgical and Dental Sciences, University of Milan, 20122 Milan, Italy; 4 Department of Pathophysiology and Transplantation, University of Milan, 20122 Milan, Italy

**Keywords:** duodenum, duodenal, pseudomelanosis, pigmentation, siderosis, iron

## Abstract

Duodenal pseudomelanosis (also known as pseudomelanosis duodeni) is a rare endoscopic incidental finding defined by a pigmentation limited to the apex of the intestinal villi, which requires histological confirmation. While its exact pathogenesis is still poorly understood, it appears free from clinical consequences. This condition is believed to be associated with oral iron intake, antihypertensive drugs containing a sulfur moiety (i.e., hydralazine, furosemide), and several chronic diseases (i.e., hypertension, end-stage renal disease, diabetes). However, the exact prevalence of these treatments and comorbidities among patients with duodenal pseudomelanosis is not clearly defined. Several case reports and case series about duodenal pseudomelanosis have been published in recent years. In this review, we aimed to clearly define its endoscopic and microscopic presentation; its epidemiology, associated comorbidities, and drugs; the most useful special histochemical techniques used to classify the nature of the pigmentation; and the most relevant differential diagnoses. In addition, by considering our findings, we also formulated a number of hypotheses about its pathogenesis.

## 1. Introduction

Duodenal pseudomelanosis (DP, Latin: pseudomelanosis duodeni) is a rare incidental finding encountered during upper gastrointestinal tract endoscopy. The usual endoscopic presentation is a black-dotted mucosa, with pigment deposition at the apex of the duodenal villi. Microscopically, the pigmentation has been reported to be composed of iron and other substances, which accumulate in lamina propria macrophages [1]. Several comorbidities and drugs have been associated with this condition, with variable prevalences across different studies. The most frequently reported associated medical conditions in the setting of DP are hypertension, end-stage renal disease, and diabetes. Intake of oral iron and antihypertensive drugs containing a sulfur moiety, such as hydralazine and furosemide, is also frequently reported to be associated with DP [2]. DP is unrelated to colonic melanosis, which is caused by chronic laxative intake and demonstrates lipofuscin deposition in lamina propria macrophages. Although a number of case reports and case series exist, a comprehensive systematic review has not been published to date in the literature, and many clinicopathological associations are not yet clearly defined. Here, we review the published literature so far, to clearly define the endoscopic and microscopic presentation of DP; its epidemiology, associated comorbidities, and drugs; and the most useful special techniques used to classify the nature of the pigmentation. In addition, we discuss the possible pathogenesis of the condition by taking into account the reports reviewed and our clustering of data.

## 2. Materials and Methods

**Figure 1:**
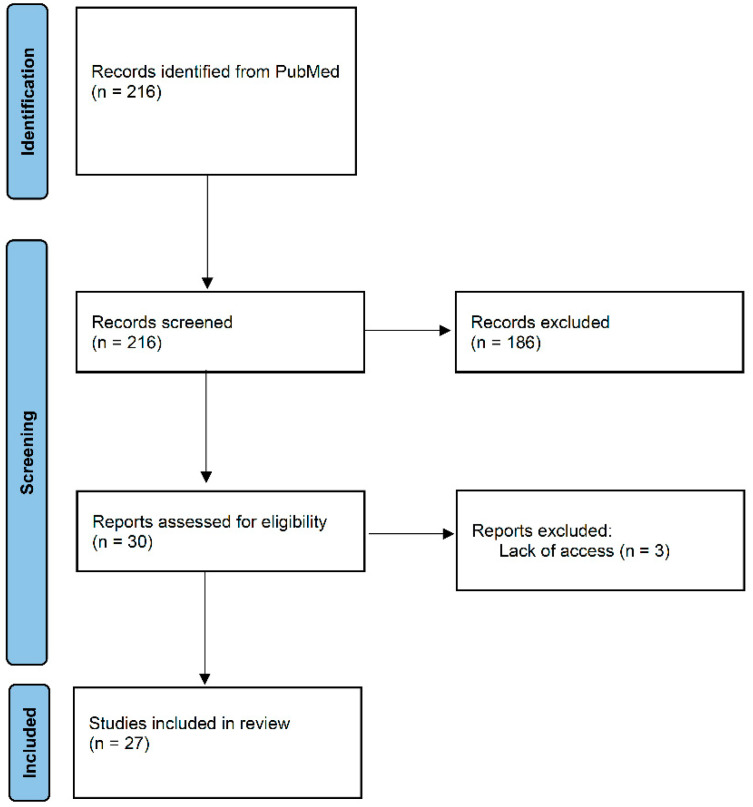
Flowchart showing identification, screening, and inclusion of studies for this review.

The online database PubMed was queried for studies published between 2001 and 2021, using the terms “duodenal pseudomelanosis”, “pseudomelanosis duodeni”, “duodenal melanosis”, “duodenal siderosis”, and “duodenal pigmentation”. In total, 216 records were identified and initially screened for inclusion. Inclusion criteria were endoscopic evidence of a DP pattern and/or microscopic evidence of pigmentation at the apex of the villi. After the initial screening, 186 studies were excluded because of a lack of relevance to the topic. A total of 30 studies remained, but 3 were excluded because of impossibility to access the full text. In total, 27 studies were included in this review. Figure 1 displays a flowchart showing the identification, screening, and inclusion of studies in this review.

## 3. Results

**Figure 2:**
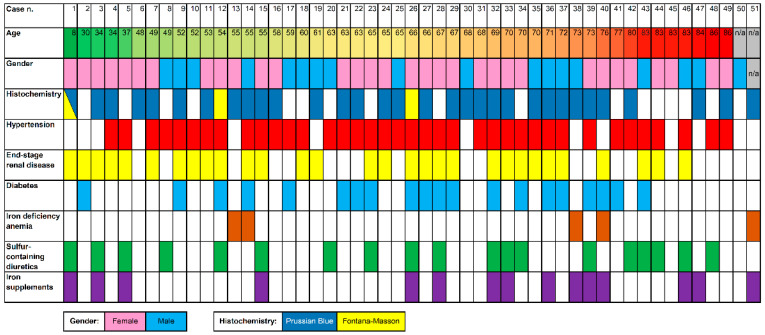
A heatmap visualization of the 51 cases of duodenal pseudomelanosis included in this review, with data on patients’ age, gender, histochemical stains positivity, relevant comorbidities, and medical treatments.

Results are presented in Table 1 and visualized in Figure 2. A total of 51 cases of DP were identified. Among these, 18 cases were male and 32 were female (F:M ratio: 1.8). A single case did not specify the patient’s gender. Patients’ mean age was 62.9 years (range 8–86 years). Two cases did not report the patients’ age.

A total of 38/51 (74.5%) patients had hypertension, 30/51 (58.8%) had renal disease, 20/51 (39.2%) had diabetes, and 5/51 (9.8%) had iron deficiency.

Moreover, 19/51 (37.2%) patients were under treatment with sulfur-containing diuretics (hydralazine, furosemide, hydrochlorothiazide), and 15/51 (29.4%) with iron supplementation; 10/51 (19.6%) patients were taking both types of medications simultaneously.

A total of 41/51 (80.4%) cases had endoscopic evidence of duodenal pigmentation. All cases had histological confirmation of macrophages within the lamina propria containing pigmented granules. In addition, 45/51 (88.2%) cases underwent special stains; 32/45 (71.1%) were positive for Prussian blue, and 3/45 (6.7%) were positive with Fontana-Masson stain. A single case was positive for both stains (1/45, 2.2%).

## 4. Discussion

The term “melanosis” is defined as an “excessive pigmentation of part of the body owing to a disturbance in melanin pigmentation” in Dorland’s Illustrated Medical Dictionary [28]. The term “pseudomelanosis” should therefore be applied to pigmentations that may resemble melanin deposition but with the demonstration of a different type of underlying pigment [9,29]. The two terms are not synonyms, as true melanosis in different organ sites refers to different clinical settings and has different implications [30,31]; however, they are widely used as synonyms in cases of diffuse pigmentation of the colon associated with excessive laxative use, with deposition of lipofuscin (melanosis coli) [32]. In practical terms, DP refers to a distinct endoscopic presentation, with pigmentation limited at the apex of the villi. The term “duodenal siderosis” could be appropriate for cases in which iron pigment is demonstrated [15].

The most common comorbidity associated with DP is hypertension, followed by renal disease, diabetes, and iron deficiency. The most frequently associated medications are sulfur-containing diuretics. The most widely adopted histochemical methods for the characterization of the pigment in the cases surveyed were Perl’s Prussian blue, which stains ferric iron (Fe^3+^), and Fontana-Masson, which stains ferrous iron (Fe^2+^) [22,33]. Among cases that underwent special stains, 34/41 (75.6%) stained positive for either Prussian Blue and/or Fontana-Masson. This demonstrates that the majority of the pigmentation in the setting of DP is due to iron deposition. The main use of Fontana-Masson stain in routine histochemistry is to demonstrate melanin deposition, but none of the Fontana-Masson-positive cases included in this review were interpreted as melanin [19,21]. A single case stained positive for both Perl’s Prussian blue and Fontana-Masson [25]. A single study analyzed a case using X-ray spectroscopy and elemental mapping, which demonstrated the presence of iron and sulfur [12]. A subset of cases (*n* = 4) focally stained positive for calcium [26].

The association of iron supplementation and DP, albeit widely stated across studies, was not completely evident in our review: only a subset of patients was under iron-pill supplementation (29.4%). In the largest case series to date (*n* = 17), a survey of duodenal biopsies of age-matched controls with a history of oral iron intake and without hypertension, diabetes mellitus, or end-stage renal disease found no evidence of iron pigmentation [26]. Given those facts, it is plausible to hypothesize that iron supplementation contributes to the pathogenesis of DP, but it is not sufficient as a single factor to induce duodenal pigmentation. The interplay between iron and sulfur may be important in this scenario, given the fact that the pigmented granules have been demonstrated to be most frequently composed of ferrous sulfate [12]. However, the proportion of patients taking sulfur-containing diuretics was also lower than expected (37.2%), as was the proportion of patients taking both medications simultaneously (19.6%). It must be noted that several studies included in this review did not state the medications taken by the patients at the time of diagnosis. When taking into account only the studies with information about treatment at the time of endoscopy (28/51, 54.9%), the proportion of patients undergoing treatment with sulfur-containing diuretics and iron supplements increased (19/28 (67.6%) and 15/28 (53.6%), respectively; 10/28 (35.7%) took both medications simultaneously). On one hand, those findings corroborate the hypothesis of a major role of these two types of medications in the pathogenesis of DP; however, other factors must be considered in order to explain DP in patients that are not under those medical treatments.

**Figure 3:**
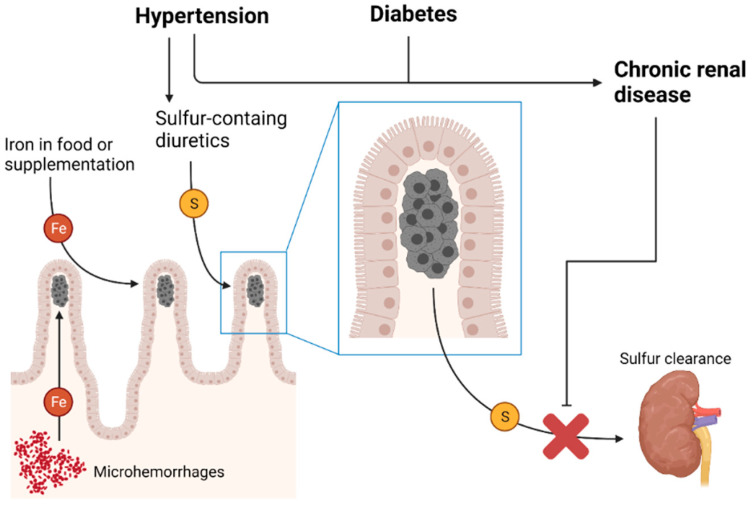
An overview of the possible pathogenesis of duodenal pseudomelanosis. The macrophages in the lamina propria at the apex of the villi contain, in most cases, sulfur (S), which is contained in some diuretics, and iron (Fe), which could accumulate due to binging with sulfur after absorption or microhemorrahgic events. Chronic renal disease causes reduced sulfur clearance, contributing to its accumulation. (Created with BioRender.com).

Dialysis, in the setting of end-stage renal disease, has been associated with the accumulation of lanthanum in the gastrointestinal mucosa [34]; a similar effect could play a role in duodenal sulfur deposition, possibly even in patients not treated with sulfur-containing diuretics. It is possible that the intake of sulfur and/or iron, contained in drugs and/or food, results in localized accumulation in the site of absorption only in the setting of an impairment of renal function and clearance. Notably, hypertension is a risk factor for chronic heart disease, commonly treated with sulfur-containing diuretics such as furosemide, and chronic renal disease; therefore, there is an indirect link between increased sulfur intake and impaired clearance. Microhemorrhagic events have also been theorized to be involved in the pathogenesis of DP [25]. It has been hypothesized that macrophages in the gastric lamina propria could be exposed to pigments via an iron-pill-induced mucosal injury [7], which was also reported in the duodenum [5]. However, all cases of DP reviewed showed no degree of acute or chronic inflammation. The pigment accumulation at the tip of the duodenal villi supports an iron-absorption-related mechanism. An overview of the possible mechanisms involved in the pathogenesis of DP is presented in Figure 3.

Differential diagnoses include iron pill duodenitis (superficial iron deposition associated with erosion and inflammatory infiltrate) [5], clofazimine-related pigmentation (patchy endoscopic presentation) [35,36], eosinophilic enteritis (focal pigmentation, eosinophilic infiltrate within Brunner’s glands) [37], and primary and metastatic melanoma (pigmented tumoral masses) [38,39]. All of these conditions harbor a worse prognosis than DP to various degrees; histopathological and histochemical examinations are thus essential tools for the formulation of a correct diagnosis.

## 5. Conclusions

DP represents a benign incidental finding caused by pigment deposition (mainly iron) at the apex of duodenal villi and is associated with certain medical conditions (hypertension, diabetes mellitus, chronic renal disease) and related therapies (sulfur-containing diuretics), which may be implicated in the pigment deposition and accumulation (duodenal drugs and iron absorption, microhemorrhages, decreased renal function). Differential diagnoses of duodenal pigmentations include diseases such as true melanosis, iron pill duodenitis, clofazimine-related pigmentation, eosinophilic enteritis, and primary and metastatic melanoma, all of which harbor different endoscopic, histologic, and histochemical findings. Pathological examination of duodenal biopsies in the setting of duodenal pigmentation is a reliable standard for diagnosis.

## Figures and Tables

**Table 1:** Summary of the studies published between 2001 and 2021 describing cases of duodenal pseudomelanosis and siderosis.

Study		Endoscopy Reason	Patient’s Gender	Patient’s Age	Patient’s History	Medications	Special Studies	Other Relevant Info
Cook D, Napthali K, 2020 [3]		Dysphagia	M	83	Chronic kidney disease, hypertension, type 2 diabetes mellitus	Hydralazine, irbesartan, lercanidipine, and metoprolol	No	
Kudaravalli P et al., 2020 [4]		Gastrointestinal bleeding	F	83	Hypertension, chronic kidney disease, hypothyroidism, atrial fibrillation	Aspirin, furosemide, metoprolol, levothyroxine, and warfarin	Yes (not published)	
Jeung J et al., 2020 [5]		Early satiety, weight loss	F	55	Iron deficiency anemia	Ferrous sulfate	Prussian blue	Pigmentation not evident at endoscopy, Brunner gland cyst, active inflammation, erosion, gastric foveolar metaplasia, reactive mucin loss
Nakanishi Y et al., 2019 [6]		Possible Crohn’s disease	M	72	Proctocolectomy with end ileostomy, chronic kidney disease, hypertension, diabetes, coronary artery disease, gout, arthritis	Not stated	Prussian blue	
Tang SJ et al., 2019 [7]		Not specified	Not stated	Not stated	Iron pill–induced mucositis	Iron supplementation	Prussian blue	Concomitant gastric pseudomelanosis
Sutaria A, Bhutani MS, 2018 [8]		Epigastric pain	F	77	Hypertension, hyperlipidemia, diabetes mellitus, hypothyroidism	Not stated	No	
Sunkara T et al., 2018 [9]		Determination of bleeding risk	F	83	Atrial fibrillation, chronic obstructive pulmonary disease, pulmonary hypertension, chronic heart failure, non-Hodgkin’s lymphoma	Rivaroxaban (discontinued)	No	Concomitant pseudomelanosis gastri
Shimamura Y et al., 2018 [10]		Gastrointestinal bleeding	M	68	Not stated	Not stated	Prussian blue	
Mundi I et al., 2017 [11]		Suspect celiac disease	M	61	Chronic renal failure	Not stated	Prussian blue	
Iwamuro M et al., 2017 [12]		Screening	M	83	Hypertension, chronic kidney disease, chronic heart failure, and chronic myeloid leukemia	Ferrous citrate, furosemide, spironolactone, tolvaptan, bisoprolol, nicorandil, warfarin, nilotinib, febuxostat, esomeprazole, digestive enzyme complex, ambroxol, carbocysteine, potassium L-aspartate	X-ray spectroscopy, elemental mapping	
Abdelwareth A et al., 2016 [13]		Dyspepsia	M	73	COPD, ischemic heart disease, Iron deficiency anaemia, colonic polyp	Esmoeprazole, ranitidine, atorvastain, losartan, inhalers, and ferrous sulfate	Prussian blue	
Coelho R et al., 2016 [14]		Dysphagia	F	76	Diabetes mellitus, chronic renal failure, iron deficiency anemia	Ferrous sulfate	Prussian blue	Schatzki’s ring
Kothadia JP et, 2016 [15]		Anemia, weight loss	M	84	Not stated	Not stated	Prussian blue	Duodenal polyp, duodenitis
Tsai YN et al., 2016 [1]		Acid reflux	M	63	Hypertension	Hydralazine	No	
Cochet AE et al., 2015 [16]		Diarrhea	M	Not stated	Not stated	Not stated	No	*Strongyloides stercoralis* infection
Sathyamurthy A et al., 2015 [17]		Intractable nausea and vomiting	M	55	Coronary artery disease, chronic kidney disease stage 4, diabetes mellitus, hypertension, iron deficiency anemia	Antihypertensives (not specified)	Prussian blue	
Siderits R et al., 2014 [18]		Abdominal pain	F	80	Hypertension	Hydralazine, laxatives (senna)	Prussian blue	
Kim SJ et al., 2013 [2]	1	Not specified	F	73	Diabetes mellitus, hypertension	Iron sulfide, antidiabetics, loop diuretics, angiotensin II receptor blockers, calcium channel blockers (not specified)	Prussian blue	
	2	Not specified	M	71	Diabetes mellitus, hypertension, chronic renal failure	Iron sulfide, antidiabetics, calcium channel blockers, potassium binders (not specified)	Prussian blue	
	3	Not specified	F	70	Hypertension, chronic renal failure	Iron sulfide, loop diuretics, angiotensin II receptor blockers, beta-blockers, statins (not specified)	Prussian blue	
	4	Not specified	F	34	Chronic renal failure	Iron sulfide, loop diuretics, statins, potassium binders (not specified)	Prussian blue	
	5	Not specified	F	69	Diabetes mellitus, hypertension, chronic renal failure	Iron sulfide, antidiabetics, loop diuretics, angiotensin II receptor blockers, calcium channel blockers, beta-blockers, potassium binders (not specified)	Prussian blue	
	6	Not specified	F	55	Hypertension, chronic renal failure	Iron sulfide, loop diuretics, statins (not specified)	Prussian blue	
Schuerle T et al., 2013 [19]		Nausea, vomiting and diarrhoea	F	54	Diabetes mellitus type 2, end-stage renal disease, kidney transplant, hypertension, anaemia, hypothyroidism	Insulin, pantoprazole, hydralazine, losartan, diltiazem, paroxetine, thyroxine, atorvastatin, cyclosporine, mycophenolate mofetil, trimethoprim/sulfamethoxazole	Fontana-Masson: positive; PAS: negative; Prussian blue: negative	
Jain SS et al., 2012 [20]		Epigastric pain	F	48	None	Proton pump inhibitor (not specified)	Prussian blue: positive; Fontana Masson: negative	
de Magalhães Costa MH et al., 2012 [21]	1	Melena, anemia	F	66	Diabetes mellitus, hypertension, chronic renal failure	Angiotensin-converting enzyme inhibitors (not specified), furosemide, ferrous sulfate, folic acid and insulin	Fontana-Masson: positive (interpreted as iron)	
	2	Acid reflux	F	37	Hypertensive nephropathy, renal transplantation	Furosemide, propranolol, hydralazine, ferrous sulfate	Not stated	
	3	Anemia	F	70	Diabetes, hypertension, chronic renal failure, nephrolitiasis, nephrectomy	Calcium channel blockers (not specified), propranolol, α-methyldopa, furosemide, glibenclamide	Not stated	
	4	Epigastric pain, nausea, vomiting	F	30	Diabetes, renal and pancreatic transplantation	Insulin, tracolimus, mycophenolate and corticosteroids (not specified)	Not stated	
Felipe-Silva A et al., 2011 [22]		Suspect upper gastrointestinal bleeding	F	86	Hypertension, ischemic strokes, left hemiplegia, central VII nerve palsy	Hydralazine, captopril, hydrochlorthiazide, aspirin	Prussian blue: negative; Fontana-Masson: negative	Distal esophagitis, gastritis and gastric angiodysplasia
Kakati B et al., 2011 [23]		Anemia	F	65	End-stage renal disease, diabetes mellitus, hypertension, hypothyroidism	Zolpidem, aspirin, clonidine, hydralazine, insulin, levothyroxine, and metoprolol	Not specified	
Yun L, 2010 [24]		Melena	F	67	End stage renal disease, coronary artery disease, diabetes, hypertension, hyperlipidemia, anemia, hypothyroidism	Aspirin, clopidogrel, hydralazine, frusemide, benazepril, atorvastatin, ezetimibe, levothyroxine, and iron	No	
Monajemzadeh M et al., 2008 [25]		Abdominal discomfort	F	8	Polycystic kidney disease, nephrotic syndrome, endstage renal disease, kidney transplant	Hydralazine, clonidine, amilodipine, oral iron supplements, phenytoin	Prussian blue: positive; Fontana Masson: positive	
Giusto D, Jakate S, 2008 [26]	1	Not specified	F	86	Hypertension	Not specified	Prussian blue: positive	
	2	Not specified	F	65	Hypertension, end stage renal disease	Not specified	Prussian blue: partial	
	3	Not specified	F	63	Hypertension, diabetes	Not specified	Prussian blue: partial	No evidence of mucosal pigmentation at endoscopy
	4	Not specified	F	68	Hypertension	Not specified	Prussian blue: partial	No evidence of mucosal pigmentation at endoscopy
	5	Not specified	M	60	Hypertension, end stage renal disease	Not specified	Prussian blue: negative	
	6	Not specified	F	63	Hypertension, diabetes	Not specified	Prussian blue: positive	No evidence of mucosal pigmentation at endoscopy
	7	Not specified	F	66	Hypertension, end stage renal disease, diabetes	Not specified	Prussian blue: partial	
	8	Not specified	M	70	Hypertension, end stage renal disease	Not specified	Prussian blue: partial	No evidence of mucosal pigmentation at endoscopy
	9	Not specified	F	34	Hypertension, end stage renal disease	Not specified	Prussian blue: partial	No evidence of mucosal pigmentation at endoscopy
	10	Not specified	M	52	Hypertension, end stage renal disease, diabetes	Not specified	Prussian blue: partial	
	11	Not specified	M	59	Hypertension, diabetes	Not specified	Prussian blue: negative	No evidence of mucosal pigmentation at endoscopy
	12	Not specified	F	53	Hypertension, end stage renal disease	Not specified	Prussian blue: partial	No evidence of mucosal pigmentation at endoscopy
	13	Not specified	M	65	Hypertension	Not specified	Prussian blue: partial	No evidence of mucosal pigmentation at endoscopy
	14	Not specified	M	52	Hypertension, end stage renal disease	Not specified	Prussian blue: negative	No evidence of mucosal pigmentation at endoscopy
	15	Not specified	F	49	Hypertension, end stage renal disease	Not specified	Prussian blue: partial	
	16	Not specified	F	58	Hypertension	Not specified	Prussian blue: positive	
	17	Not specified	F	67	Hypertension, end stage renal disease, diabetes	Not specified	Prussian blue: partial	No evidence of mucosal pigmentation at endoscopy
Cantu JA, Adler DG, 2005 [27]		Jaundice after cholecystectomy	M	49	Chronic renal insufficiency, hypertension	Hydralazine	Not stated	
